# The Relationship between Neovascular Age-Related Macular Degeneration and Erectile Dysfunction

**DOI:** 10.1155/2013/589274

**Published:** 2013-09-26

**Authors:** Harun Çakmak, Tolga Kocatürk, Sema Oruç Dündar, Mehmet Dündar, Müjdat Karabulut

**Affiliations:** ^1^Department of Ophthalmology, Adnan Menderes University Medical Faculty, Merkez Kampus Kepez Mevkii, Aytepe, 09100 Aydın, Turkey; ^2^Department of Urology, Adnan Menderes University Medical Faculty, Aydın, Turkey

## Abstract

*Purpose*. To evaluate association between erectile dysfunction (ED) and neovascular age-related macular degeneration (AMD). *Methods*. 195 men enrolled in this cross-sectional study. 90 of them had neovascular AMD and 105 of them were healthy volunteers. The International Index of Erectile Function (IIEF) questionnaire's erectile function (EF) domain was used to assess ED. The patients in the study and control groups were statistically compared according to visual acuity, EF score, and body mass index. *Results*. The mean ages were 62 (54.5–73) and 60 (54–68), in the neovascular AMD and control groups, respectively. The total EF scores were 9 (6–16) in neovascular AMD and 18 (9.5–27) in control group. The results of IIEF questionnaire on neovascular AMD patients revealed that 85 men (94.4%) had some degree of ED, whereas 68 men (64.8%) had some degree of ED on control group. Patients with neovascular AMD had a significantly higher incidence of ED than control patients (*P* < 0.01). There was a significant association between ED and neovascular AMD (*P* < 0.01). *Conclusions*. Our results suggested that neovascular AMD has a high association with ED.

## 1. Introduction

 Age-related macular degeneration (AMD) is the most common cause of blindness in industrialized countries affecting individuals over the age of 55 [1]. Neovascular AMD affects only 10–15% of AMD patients but approximately 90% of blindness due to this condition [2]. The main cause of vision loss in neovascular AMD is the development of choroidal neovascularization, which is ultimately the result of a break in a structural layer beneath the retina known as Bruch's membrane, which separates the nourishing vascular layer called the choroid from the retina. These vessels can leak fluid or blood, initially distorting or blurring vision, and may eventually lead to scar in the macula and severe loss of central vision [3].

 Erectile dysfunction (ED) is defined as the inability to attain or maintain the penile erection required for sufficient sexual performance [4]. ED is a common health problem in middle aged and elderly men [5]. The prevalence of this dysfunction increases steadily from 10% to 80% with age [6]. ED is a multifactorial disease; the vascular, neurogenic, hormonal, psychogenic, cavernosal, iatrogenic, and anatomic causes lie in its pathophysiology. ED can affect emotional, sexual, social, recreational, and intellectual intimacy [7]. The impact of ED can be devastating because evidence has shown that sexual function is one of the very important indices of quality of life [8].

 Cross-sectional studies have reported that ED is strongly associated with diabetes mellitus, hypertension, cardiovascular disease, hyperlipidemia, metabolic syndrome, depression, and lower urinary track symptoms [9–15]. Medication and recreational drugs are also associated with male sexual disorders, especially the use of antipsychotic drugs [16]. New epidemiological studies show an increase in the prevalence rates for ED in men who smoke as a standalone risk [17]. Also, obesity, low testosterone levels, poor quality of life, apnoea-hypopnoea, and obstructive sleep apnoea syndrome were significantly associated with a higher risk of ED [18].

To the best our knowledge, the association between neovascular AMD and ED has not been evaluated before. In this cross-sectional study, we aim to investigate the relationship between ED and neovascular AMD.

## 2. Material and Methods

 This prospective, cross-sectional study was conducted at the ophthalmology clinic between December 2012 and March 2013. The study was conducted according to the Declaration of Helsinki, the European Guidelines on Good Clinical Practice, and relevant national and regional authority requirements and the university's ethics committees. All subjects signed informed consent forms before participating in the study. 

 Ninety sexually active men with neovascular AMD (Group 1), who presented to our clinic, enrolled in the study. The control group (Group 2) was formed with the participation of 105 randomly chosen healthy volunteers from among those in the outpatient clinic. Both groups included married men who had been sexually active.

Patients with diabetes mellitus, hypertension, hyperlipidemia, heart disease, or other known systemic diseases (such as depression, stroke, and medications) and/or smokers were excluded. 

 The International Index of Erectile Function (IIEF) is a widely used, multidimensional self-report instrument for the evaluation of male sexual function [19]. The presence and severity of ED were determined by calculating the EF domain of the questionnaire (EF score). Because we aimed to evaluate EF, we used IIEF's EF domain. The EF domain includes six questions. The responses to all the six questions of the EF domain were added to arrive at a total EF score, with a range from six to 30. The six items on the EF domain include detailed questions concerning erection frequency, erection firmness, penetration ability, maintenance frequency, maintenance ability, and erection confidence [20]. A higher total score indicated relatively better erectile functioning. ED severity was rated as no ED if scored from 26 to 30, mild ED if scored from 17 to 25, moderate ED if scored from 11 to 16, and severe ED if scored from 6 to 10. 

 All subjects had undergone detailed ophthalmologic examination. Best corrected visual acuity of each eye and binocular vision were evaluated. We separated the patients according to their binocular vision (low vision or not) and patients were divided into two groups with or without binocular vision higher than 0.3. Biomicroscopic anterior segment and fundoscopic retinal examination were done. AMD was diagnosed with fundoscopic retinal examination, fundus fluorescein angiography, and optical coherence tomography. 

 Data analysis was performed with SPSS (Statistical Package for Social Sciences), version 17.0. The Kolmogorov-Smirnov test was used to assess the normality of numeric variables. For the normally distributed variables, comparison between the two groups was made by the independent sample *t* test and one-way ANOVA, and the results were expressed as mean ± standard deviation. For the nonnormally distributed variables, comparison between two groups was made by the Mann Whitney *U*-test and the descriptive statistics were expressed as median (25–75 percentiles). Chi-square test was used to analyse nominal data. Significance was defined as *P* < 0.05.

## 3. Results

 Demographic characteristics of participants for both groups are presented in Table 1. In Group 1, there were 90 men with a mean age of 62 years, ranging from 54.5 to 73. In Group 2, there were 105 men with a mean age of 60 years, ranging from 54 to 68.

**Figure 1:**
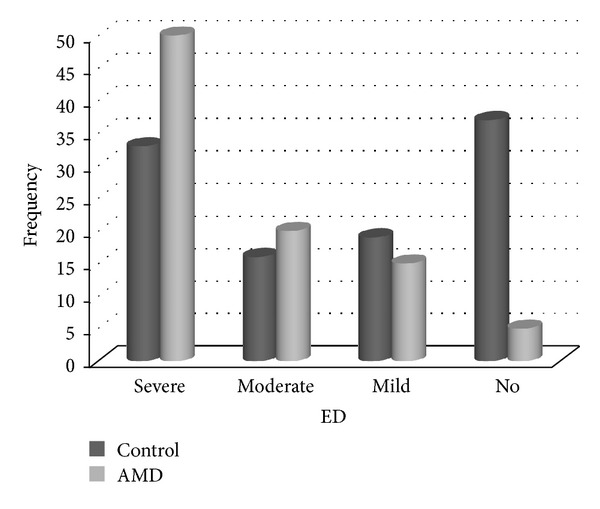
Distribution of subjects across different EF severities. AMD: age-related macular degeneration, ED: erectile dysfunction. Erectile function severities between control and AMD group were statistically significant (*P* < 0.01).

Figure 1 shows the EF in terms of IIEF score. In Group 1, the results of the EF domain score revealed that 50 patients (55.6%) had severe ED, 20 patients (22.2%) had moderate ED, 15 patients (16.7%) had mild ED, and 5 patients (5.6%) had no ED. In Group 2, the results of the patients' EF domain score revealed that 33 (31.4%) had severe ED, 16 (15.2%) had moderate ED, 19 (18.1%) had mild ED, and 37 (35.2 %) had no ED. 95% of subjects had some degree of ED in AMD group. Similarly, 88.1% of men with no ED also had no AMD.

Patients with neovascular AMD had a significantly higher incidence of ED than control patients (*P* < 0.01). There was a significant association between ED and neovascular AMD (*P* < 0.01).

**Figure 2:**
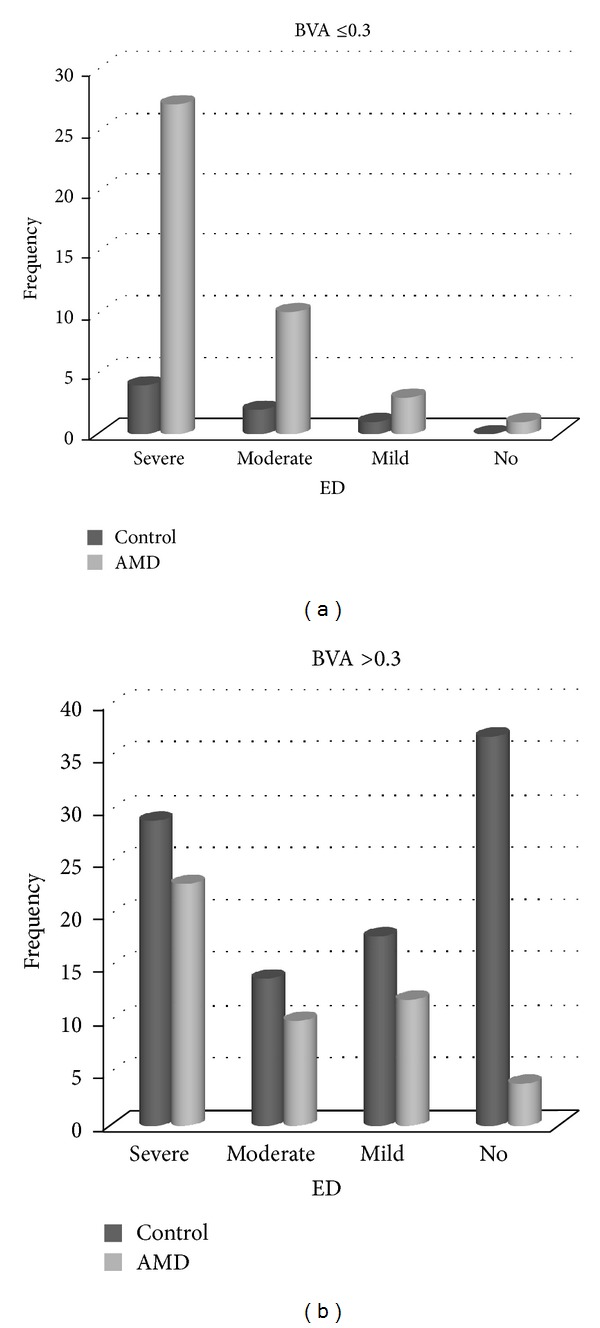
Erectile function in AMD and control group according to the binocular vision higher than 0.3 or not. BVA: binocular vision; ED: erectile dysfunction; AMD: age-related macular degeneration.

 According to binocular vision classifications, there were 49 and 98 men whose binocular vision was higher than 0.3 in Groups 1 and 2, respectively. Similarly, there were 41 and 7 men with a binocular vision of 0.3 or lower in Groups 1 and 2, respectively. In Group 1, 97.6% of the subjects with binocular vision of 0.3 or lower had ED. In Group 2, all of the participants with binocular vision of 0.3 or lower had ED. 62.2% of controls with good vision (VA > 0.3) had ED, while 91.8% of AMD patient with good vision had ED. There was no statistically significant difference according to ED and visual level in neovascular AMD group (*P* = 0.16). Conversely, in control group, the difference was statistically significant (*P* = 0.03) (Figure 2).

 BMI was calculated (kg/m^2^) and subjects were categorized as normal (22 to 24.9 kg/m^2^), overweight (25–29.9 kg/m^2^), or obese (≥30 kg/m^2^) according to their BMI values. Mean BMI was 26.71 ± 3.1 and 25.87 ± 4.1, in Groups l and 2, respectively. There were 22 normal weight men, 53 overweight men, and 15 obese men in Group 1. There were 47 normal weight men, 43 overweight men, and 15 obese men in Group 2. 93.3% of obese men, 94.7% of overweight men, and 95.5% of normal weight men were found to have ED in Group 1. 

 While in control group only 12 overweight subjects had severe ED in AMD group, 33 overweight subjects (nearly three times as many as controls) had severe ED. This difference is statistically significant (*P* < 0.01). In control group, 6 subjects had moderate ED, while in AMD group 13 subjects had moderate ED (*P* < 0.01) (Table 2).

 We separated the subjects according to their ages. In Group 1, all subjects had ED in the 4th and 5th decades. In the 6th decade and over the age of 70, 87.5% and 96.7% of patients had ED, respectively. 46.2% of patients in the 4th decade and 56.1% of patients in the 5th decade were found to have ED in Group 2. In the 6th decade and over the age of 70, 71% and 85% of patients had ED, respectively (Table 3).

## 4. Discussion

The association between neovascular AMD and ED has never been elucidated in the literature. In the present study, we examined the relationship between neovascular AMD and ED.

Risk factors of AMD and ED are mostly similar. Diabetes mellitus, hypertension, stroke, chronic kidney disease, atherosclerosis, abnormalities of lipid metabolism, hyperlipidemia, and cardiovascular disease are positively associated with AMD [21, 22]. Also, several studies have attempted to assess the relationship between ED and risk factors such as diabetes mellitus, depression, cardiovascular disease, smoking, hypertension, increased age, hypogonadism, hyperprolactinemia, and hyperparathyroidism [23]. 

In the control group among patients with no risk factors or any of the medical comorbidities, 64.8% had some degree of ED and of them 31.4% had severe ED. This prevalence and severity of ED seem to be high for controls. On the other hand, these results were found to be much more higher in neovascular AMD group. In neovascular AMD group among patients with no risk factors or any of the medical comorbidities 94.6% had some degree of ED and of them 55.6% had severe ED.

 AMD shares several pathological and epidemiological similarities with ED. Some common factors are involved in their pathological processes [24–28]. 

 First, both choroid and penis have a rich vasculature and impaired microcirculation has been disclosed in the choroids of patients with neovascular AMD and in the penises of men with ED [22–24]. Second, some recent studies have demonstrated that disorders with endothelial dysfunction are associated with ED [25]. The penis is a highly vascularized organ, and erections are primarily vascular events. Third, nitric oxide (NO) is particularly important; vasodilation is essential for erection, and NO has the trigger role for vasodilation in the vascular endothelium. Disorders with endothelial dysfunction will also affect erection by interfering vasodilation.

Vascular endothelium regulates retinal arteriolar tone and circulation in the ophthalmic and ciliary arteries. Endothelial dysfunction has a crucial role in the development of neovascular AMD [26]. Several clinical trials have shown soluble markers related to endothelial dysfunction, for example, sICAM-1. It increases in different types of AMD [27]. There is considerable evidence implicating endothelial dysfunction and cellular oxidative stress in the pathogenesis of both AMD and ED [28]. 

 A number of biological mechanisms may link obesity to sexual dysfunction. Potential mechanisms include endothelial dysfunction, metabolic syndrome and diabetes, altered endocrine function, social and psychological problems, and obstructive sleep apnoea, as well as ordinary physical disabilities. Lifestyle factors such as smoking, high alcohol intake, poor diet, and physical inactivity may also influence sexual dysfunction [29, 30]. Interestingly, these observations were not corroborated by Paick et al. who did not find a significant relationship between ED severity and metabolic syndrome, BMI, hormones, lifestyle [31]. Smith et al. studied an association between BMI and sexual satisfaction. No associations were found for either men or women between BMI and sexual satisfaction within their relationship [32]. 

 Our study revealed a statistically significant relationship between BMI and ED in control group (*P* = 0.01). We also did not find any strong association between BMI and ED in neovascular AMD group (*P* = 0.121).

 Depression is characterized by loss of interest, reduction in energy, lowered self-esteem, and inability to experience pleasure. This constellation of symptoms may be expected to produce difficulties in sexual relationships and depression has long been associated with sexual problems [33]. Several studies indicate that low vision secondary to AMD is a risk factor for depression and this has serious consequences for the quality of life among patients with AMD [34]. Depression and stress are also psychogenic factors of ED and stress-related characteristics are found in patients with AMD. Patients with more severe vision loss prior to treatment tended to report more depression. Augustin et al. found that the prevalence of depression increased as visual impairment became more severe [35]. Rates ranged from 14.3% for those with minimal vision loss to 25% for those with severe vision loss. The prevalence of depression was also high among older adults who sought low vision rehabilitation services [36]. 

 In ophthalmology practice, if binocular vision is lower than 0.3, it is called “low vision” [37]. That is why we separated patients with or without binocular vision higher than 0.3. Low vision results in depression and connected with this; depression also results in ED [33]. So, low vision may have negative effects on EF indirectly. We separated the patients according to their binocular vision (low vision or not) and we could clarify if the ED is because of AMD or low vision. In control group those patients whose binocular vision was 0.3 or lower had a significantly higher incidence of ED than patients with good binocular vision. Interestingly, in neovascular AMD group there was no association between visual levels and ED.

 Tsai et al. demonstrated epidemiological evidence of a significantly increased ratio of ED among patients with central serous chorioretinopathy (CSCR) diagnosis. They claimed that some factors like glucocorticoids, microvascular pathologies, and endothelial dysfunction prone to inducing CSCR could also have a harmful effect on EF [38]. 

 We demonstrated a significantly increased ratio of ED among patients with neovascular AMD diagnosis. There are many similar risk factors between the etiology and pathogenesis of AMD and ED. To the best of our knowledge, this study is the first study that shows the association between neovascular AMD and ED. Further studies are warranted to explore the underlying mechanism of this relationship.

## Figures and Tables

**Table 1:** Characteristics of the 195 study subjects included in this study.

	AMD group (*n* = 90)	Control group (*n* = 105)	*P*
Age	62 (54.5–73)	60 (54–68)	0.054
Height	1.70 (1.67–1.72)	1.70 (1.65–1.74)	0.352
Weight	75 (70–82)	74 (65–82)	0.119
BMI	26.7 ± 3.1	25.9 ± 4.1	0.106
BVA	0.5 (0.2–0.7)	1.0 (0.8–1.0)	<0.001
Total IIEF-EF domain score	9 (6–16)	18 (9.5–27)	<0.001

BMI: body mass index; BVA: binocular visual acuity; IIEF-EF: International Index of Erectile Function-Erectile Function.

**Table 2:** Erectile function in AMD and control group according to BMI.

IIEF-EF domain	AMD group no. (%)			Control group no. (%)		
	Normal	Overweight	Obese	Normal	Overweight	Obese
Severe ED	9 (40.9)	33 (62.3)*	8 (53.3)	10 (21.2)	12 (27.9)*	9 (60)
Moderate ED	6 (27.3)	13 (24.5)^†^	1 (6.7)	8 (17.1)	6 (14.0)^†^	3 (20)
Mild ED	6 (27.3)	4 (7.5)	5 (33.3)	11 (23.4)	8 (18.6)	1 (6.7)
No ED	1 (4.5)^‡^	3 (5.7)^‡^	1 (6.7)^‡^	18 (38.3)^‡^	17 (39.5)^‡^	2 (13.3)^‡^

Total	22 (100)	53 (100)	15 (100)	47 (100)	43 (100)	15 (100)

BMI: body mass index, ED: erectile dysfunction, AMD: age-related macular degeneration.

Percentages are shown in parantheses.

^∗,†^In AMD overweight subgroup it could be seen that subjects who have severe and moderate ED are more than those in control overweight subgroup.

^‡^In control group, independently of BMI values, more subjects who do not have ED could be seen.

**Table 3:** Erectile function in AMD and control group according to the ages.

Group	Age	Severe ED	Moderate ED	Mild ED	No ED	Total
AMD (*n* = 90)	40–50	10	8	0	0	18
	51–60	5	4	1	0	10
	61–70	16	5	7	4	32
	>70	19	3	7	1	30

Control (*n* = 105)	40–50	2	1	3	7	13
	51–60	9	6	8	18	41
	61–70	10	7	5	9	31
	>70	12	2	3	3	20

Total		83	36	34	42	195

ED: erectile dysfunction, AMD: age-related macular degeneration.
